# Soluble HLA peptidome: A new resource for cancer biomarkers

**DOI:** 10.3389/fonc.2022.1069635

**Published:** 2022-12-22

**Authors:** Erwin Tanuwidjaya, Ralf B. Schittenhelm, Pouya Faridi

**Affiliations:** ^1^ Monash Proteomics & Metabolomics Facility, Department of Biochemistry and Molecular Biology, Monash Biomedicine Discovery Institute, Monash University, Clayton, VIC, Australia; ^2^ Department of Medicine, School of Clinical Sciences, Monash University, Clayton, VIC, Australia

**Keywords:** cancer biomarkers, liquid biopsy, immunopeptidomics, mass spectrometry, soluble HLA, HLA peptidome

## Abstract

Using circulating molecular biomarkers to screen for cancer and other debilitating disorders in a high-throughput and low-cost fashion is becoming increasingly attractive in medicine. One major limitation of investigating protein biomarkers in body fluids is that only one-fourth of the entire proteome can be routinely detected in these fluids. In contrast, Human Leukocyte Antigen (HLA) presents peptides from the entire proteome on the cell surface. While peptide-HLA complexes are predominantly membrane-bound, a fraction of HLA molecules is released into body fluids which is referred to as soluble HLAs (sHLAs). As such peptides bound by sHLA molecules represent the entire proteome of their cells/tissues of origin and more importantly, recent advances in mass spectrometry-based technologies have allowed for accurate determination of these peptides. In this perspective, we discuss the current understanding of sHLA-peptide complexes in the context of cancer, and their potential as a novel, relatively untapped repertoire for cancer biomarkers. We also review the currently available tools to detect and quantify these circulating biomarkers, and we discuss the challenges and future perspectives of implementing sHLA biomarkers in a clinical setting.

## Introduction

The pursuit of cancer biomarker discovery serves the purpose of identifying, characterizing, and monitoring specific molecules (or entire cells) in patients, allowing for early detection of cancer and/or its differentiation from non-cancerous tissue. There are three main types of biomarkers: 1) diagnostic, 2) prognostic and 3) predictive biomarkers (1). A diagnostic biomarker refers to a marker that allows for the detection and identification of a particular type of cancer. In contrast, a prognostic biomarker offers information on the likelihood of survival as well as on potential future disease progression, while a predictive biomarker informs clinicians and physicians about appropriate and suitable therapeutic treatments (1). Irrespective of the type of biomarker, an ideal marker should be capable of reproducibly and robustly discriminating healthy individuals from patients (or subsequently the progress of diseases). In addition, it should be easy and inexpensive to assay with the possibility of high-throughput screening (2). Various types of biomarkers including cancer biomarkers have been extensively reviewed (1, 3), and the discovery and utilities of some of the most well-known cancer biomarkers have been discussed in great detail elsewhere (4).

Molecular profiling techniques to identify or screen biomarkers are typically initiated by acquiring tumor samples from patients using invasive surgeries, often referred to as tissue biopsies. Moreover, repeated surgeries are often required to observe the development of a specific tumor over time, especially in the case of metastasis (5). The cost of these surgeries in combination with the risk of conducting repeated invasive procedures on the same patient has created a pressing need for alternative methods to identify and screen biomarkers. One attractive solution is the use of liquid biopsies, which refers to a collection of techniques developed to detect, quantify and characterize circulating tumor cells (6) and/or cancer-related molecules in various, easily accessible body fluids. Measurement and analysis of biomarkers in such liquid biopsies allow for the prediction of cancer pathogenic processes *via* tumor-specific alterations in cancer (7–9).

Body fluids are fluids produced by the body for 1) normal bodily functions (e.g. blood), 2) as waste products (e.g. urine), or 3) in disease pathology (e.g. malignant pleural effusion, which results in the accumulation of exudative fluid in the lungs due to pathology-induced fluid imbalances) (10). The most commonly studied body fluid for human biomarker discovery is blood, which is readily accessible and contains circulating molecules from all over the body, including proteins and other biomolecules originating from the tumor(s). As such, blood – and its processed derivatives plasma and serum – are an attractive source for biomarker discovery translational research. Blood, however, contains a huge dynamic range of protein concentrations (13 orders of magnitude) with over half of the total protein mass made up of albumin (11). These highly abundant proteins complicate the detection of cancer-associated proteins, which are expected to be low in abundance. As of 2021, a total of 5,877 plasma proteins have been cataloged through the Human Plasma Proteome Project (HPPP) (12). This number, however, represents only one-fourth of the approximately 20,000 proteins characterized and annotated in the human proteome (13). Therefore characterizing protein biomarkers from blood will likely not provide a comprehensive view of the entire proteome expressed in tumors. Research on biomarker discovery is not only limited to blood. Cancer-associated proteins can also be present in other body fluids such as pleural effusion (14), saliva (15) and urine (16). For instance, soluble mesothelin-related peptide, which is an FDA-approved biomarker for clinical use in mesothelioma, has a high abundance in malignant pleural effusion (14). However, in contrast to blood, which contains proteins from all body organs, these body fluids predominantly contain proteins that are only released locally.

One additional major limitation of using proteins present in any body fluid as a source for biomarkers is that the array of proteins, which are present in these fluids, originated mostly from secreted and membrane-bound proteins. These two classes of proteins only represent around 35% of the whole proteome (17, 18). More importantly, only ~6% of known cancer-associated proteins are confirmed or predicted as secretory proteins and thus, cannot be detected in the intact form in body fluids (**Supplementary Table S1**) (19). As such, there is a clear need for alternative approaches if we want to use body fluids as a means to identify tumor-specific protein biomarkers.

Human Leukocyte Antigen (HLA) is a protein-product of the Major Histocompatibility Complex (MHC) gene in Humans and presents peptides on the cell surface for T cell recognition. The cargo of peptides bound to HLA proteins is termed the immunopeptidome (20). The immunopeptidome plays an essential role in immunomodulation as presented peptides may belong to either self or non-self (21). There are two main classes of HLA: 1) HLA class I (HLA-I) and 2) HLA class II (HLA-II). HLA-Is are expressed in the majority of nucleated cells and present peptides (typically between 8 to 12 amino acids long) from inside the cell, which are produced from proteasomal degradation (22). HLA-II molecules bind longer peptides (between 13 to 20 amino acids) and are expressed mostly by antigen presenting cells (APCs) (23). The peptide cargoes of HLA-II peptides are mostly of extracellular origin. As such, the collective immunopeptidome of HLA-I and HLA-II is not limited to secretory proteins, but rather represents snapshots of entire cellular proteomes including their surroundings.

Both HLA-I and HLA-II are predominantly membrane-bound (often termed mHLA), but intriguingly, a fraction of HLA molecules is released into body fluids where they are referred to as soluble HLAs (sHLAs) (24). In this review we discuss the potential to exploit the sHLA immunopeptidome of various body fluids as a novel, innovative and relatively untapped repertoire to identify circulating biomarkers derived from the entire cancer proteome. First, we broadly discuss the potential for sHLA immunopeptidome to be used as a source of cancer biomarkers including various concrete examples, and then we discuss our vision of analyzing these biomarkers at low cost in a high throughput manner in the context of translational clinical screening.

## Sourcing biomarkers from soluble HLA: Current evidence and examples

The presence of HLA proteins in human plasma was first discovered by van Rood and colleagues in 1970 (25). Three soluble forms of HLA proteins have been discovered so far (26): 1) A ~35 kDa version corresponding to the extracellular domain of an mHLA molecule (27); 2) A ~39 kDa version originating from an alternative splicing event (28); and 3) the fully shed ~44 kDa mHLA molecule (24). There is increasing evidence linking the abundance of sHLA protein in serum to disease progression and immune evasion in cancer, particularly in protection from immune recognition and inhibition of destruction of the microenvironment (29). Additional studies have examined a potential link between sHLA levels and malignancy. While the majority of these studies agreed that elevated sHLA levels correlate with poorer prognosis, a few studies suggest otherwise (24, 30). It has to be noted however that these observations differ depending on the cancer type, and could therefore be explained by different immune reactions toward various cancer types. Regardless of the abundance of sHLA, several studies confirm their presence in different body fluids (**Table 1**) (31, 32, 34, 35, 37–42). Interestingly, peptides bound to these sHLA are derived from intra- and extracellular proteins expressed in the cell of origin. As such, the sHLA immunopeptidome acts as a reservoir of all proteins expressed in a cell including peptides derived from cancer-specific proteins in tumors (45).

**Table 1:** Summary of various body fluid in which presence of HLA has been validated *via* proteomics studies.

Body fluid	Origin of proteins	No. of proteins detected in the body fluid	Coverage for tumor-associated antigen^$^	Evidence of the presence of HLA in this body fluid
Amniotic fluid	Maternal blood	3025*	<3% (90/3025)	(31)
Aqueous humor	Eyes	1888**	<3% (50/1888)	(32)
Blood	Whole body organs	5877***	<3% (157/5877)	(33)
Cerebrospinal fluid	Brain	4364*	<3% (122/4364)	(34)
Milk	Whole body organs	2457*	<3% (67/2457)	(35)
Pleural effusion	Lungs	1519*	4% (62/1519)	(36)
Saliva	Oral cavity, whole body organs	2758*	3.3% (92/2758)	(37)
Seminal fluid	Male reproductive organs	4084*	3.6% (145/4084)	(38)
Sweat	Skin, whole body organs	1244*	3.1% (39/1244)	(39)
Synovial fluid	Joints, whole body organs	1637*	3.6% (59/1637)	(40)
Tears	Eyes	1882*	3.4% (64/1882)	(41)
Urine	Urinary organs, reproductive organs	7330*	<3% (196/7330)	(42)

*taken from Human Body Fluid Proteome database (43).

**taken from the latest available MS-based proteomics study with compiled database from all existing aqueous humor proteomics studies (44).

***taken from the latest Human Plasma Proteome Project-associated study article (12).

^$^data compiled in the **Supplementary Table S1**.

This notion of exploring the sHLA immunopeptidome in the context of cancer biomarkers was initiated by Bassani-Sternberg and colleagues slightly more than a decade ago (33). In the seminal study comparing HLA immunopeptidomes in human plasma to hematological cancer cell lines, they aptly recognized that the sHLA complexes (consisting of the sHLA molecule themselves and their peptide ligands) remain stable in circulation for two days. More importantly, the peptide cargoes of the sHLAs were highly similar to those carried by their mHLA counterparts. These observations opened up the possibility of characterizing all proteins present in a tumor cell by studying peptides presented by sHLA complexes in body fluids.

At present, only a handful of studies exist describing sHLA immunopeptidomes, and many of those studies are centered around sHLA-I peptides isolated from blood (33, 46–48). The above-mentioned study by Bassani-Stenberg (33) identified a number of peptides derived from cancer-testis antigens (CTA) and tumor-associated antigens (TAA) in the blood of patients with hematological malignancies. More recent studies observed that sHLA complexes containing peptides derived from validated CTAs (macrophage inhibitory factor (49, 50) & NY-ESO-1 (51)) can be detected in the blood of breast cancer patients (52).

In 2016, Ritz and colleagues (46) conducted validation studies on the methodology for the current mHLA immunopeptidome extraction from cell lines. Once validated, the method was then implemented on the sera of melanoma patients and healthy individuals to assess its effectiveness in extracting sHLA immunopeptides. Their investigation identified a total of 22 peptides derived from validated melanoma-associated antigens (53), 15 of which were exclusively found in serum. Just one year later, the same laboratory successfully improved their sHLA peptidome characterization from a few hundred peptides in their original study (46) to about 2,000 peptides by adding an additional purification step (47). This number was further increased again to 26,841 peptides in 2019 (48), demonstrating a tremendous improvement in method development. In this study, Shraibman et al. managed to validate that the expression of potential biomarkers derived from plasma sHLA in glioblastoma patients change pre- vs post-surgical interventions, whilst most of the peptides remain constant during the same period of time (48).

In a more recent report, Khazan-Kost and colleagues discovered the potential of sHLA as a valuable source of biomarkers for lung cancers in pleural effusion (54). His work attempts to expand from the 2002 study in which Amirghofran and colleagues validated the presence of sHLA-I in malignant pleural effusion (36), to provide a comparison between malignant vs benign pleural effusions. Analysis of both the soluble and membrane HLA immunopeptidome resulted in the identification of a total of 32,970 unique HLA peptides derived from 11,305 proteins from both benign and malignant effusion with clear distinctions between them (19,294 and 1,784 peptides have been exclusively identified in the malignant and benign effusion, respectively). More importantly, unique sets of peptides derived from TAAs specific to lung adenocarcinoma were observed exclusively in pleural effusion and not in corresponding individual’s plasma samples. The protein used as an example in the study, anaplastic lymphoma kinase (ALK), is not yet validated and classified as a CTA/TAA at present (55), but this protein has a potential to be a localized biomarker for lung cancer. Finally, while the study predominantly focused on pleural effusion, it also validated that the sHLA immunopeptidome can serve as a valuable source of TAAs as evidenced by the identification of several known TAAs (SAGE1, PBK and ODF2) in both pleural effusion and plasma samples of lung cancer patients.

The investigation of the sHLA-II immunopeptidome proved to be much more challenging than the HLA-I counterpart. At present, there is little progress on the identification and characterization of the peptide cargo of sHLA-II molecules (56). Combined with the lower abundance of sHLA-II in blood it is not surprising that only ~200 peptides have been identified from 3 mL of human plasma from healthy individuals (56). There are currently no HLA-II peptidome studies of cancer patients yet, either from blood nor any other body fluid.

## Analysis of sHLA immunopeptides by using immunopeptidomics

**Figure 1:**
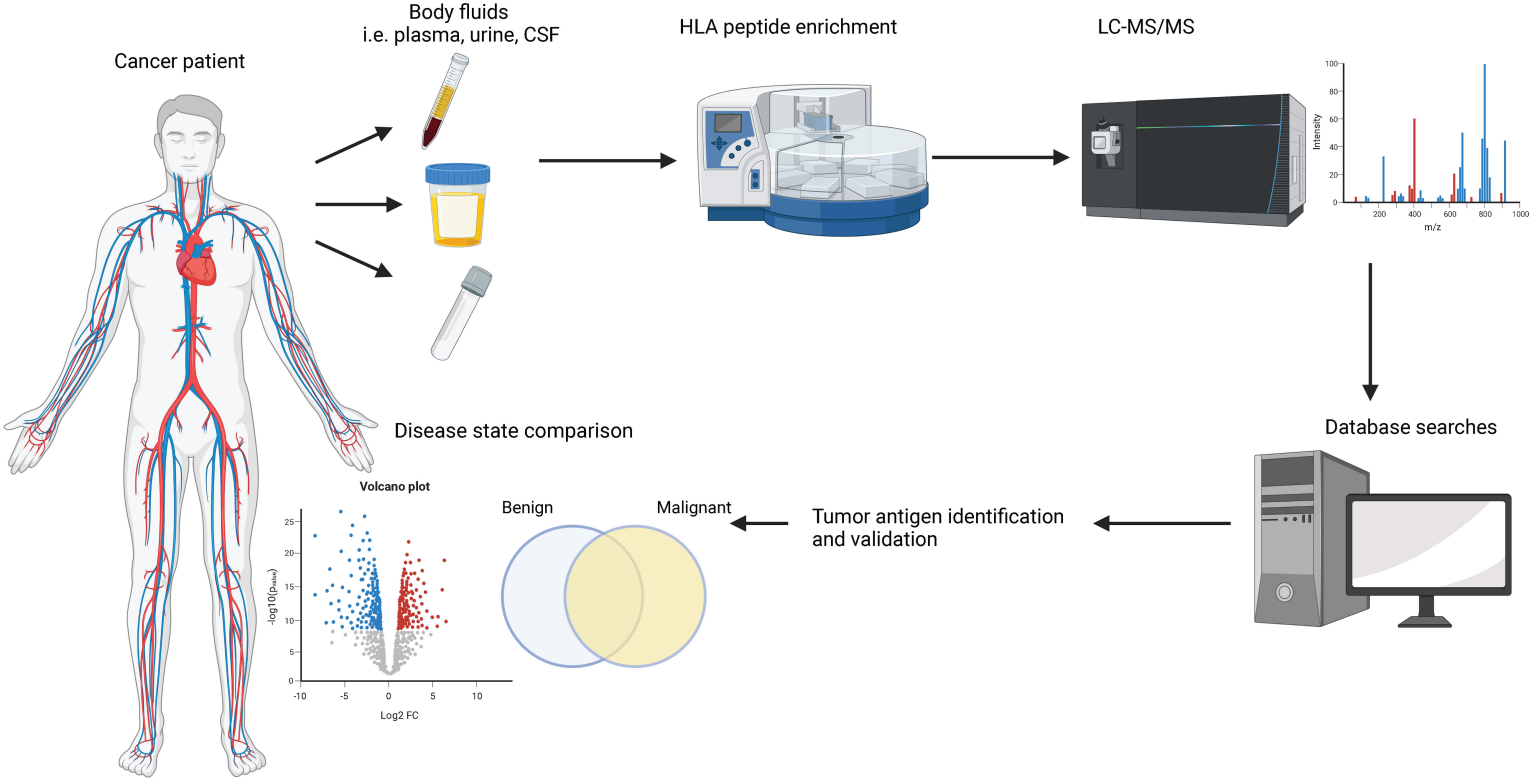
Commonly used pipeline to study HLA immunopeptidomes. Body fluids are extracted from cancer patients or healthy donors via minimally invasive procedures. sHLA-peptide complexes are enriched by immunoprecipitation assays and the peptide cargo is separated under mild acidic conditions. The peptides are analyzed by liquid chromatography-tandem mass spectrometry (LC-MS/MS) and various software packages are used to search the data against a human proteome database to obtain peptide sequence information. State-of-the-art bioinformatic analyses are employed to shortlist potential biomarkers.

Immunopeptidomics is the method of choice for the identification and quantification of sHLA-bound peptides and the pipeline is summarized in **Figure 1**. In brief, the body fluid of interest is collected from the patient *via* a minimally invasive procedure, and sHLA-peptide complexes are enriched *via* immunoaffinity purification (IP) using HLA-specific antibodies. Peptides are eluted from the sHLA protein by acidification and analyzed by liquid chromatography-tandem mass spectrometry (LC-MS/MS). The data is then analyzed against a human proteome reference database and/or a personalized database in the case of a proteogenomics approach (57). The peptides’ source proteins are then typically mapped against the cancer testis antigen database (58).

Recent advances of the immunopeptidomic pipeline have resulted in significantly increased peptide identifications (IDs) as well as data accuracy and quality. This was predominantly accomplished by mitigating issues that have been plaguing earlier HLA peptidome studies. For example, earlier studies heavily relied on the extraction of mHLA-bound peptides directly from intact cancer cell lines using mild acid elution (MAE), which is highly prone to contamination (59). The implementation of antibody-based IP enrichments of HLA-peptide complexes after cell lysis resulted in a 6-fold increase in peptide numbers compared to the earlier MAE approach (60). Similarly, significant advances in the sensitivity, selectivity and speed of mass spectrometric instrumentation have led in the past 10 years to an increase in peptide IDs that can be identified from decreasing amounts of starting material (61). Just a decade ago, 10^10^ cells were required to identify approximately 3,000 peptides (62). A similar number of peptides was identified with 100-fold less starting material (10^8^ cells) just a few years later (63) and most recently, with as little as 10^7^ cells (64). In the context of liquid biopsy analysis, current approaches have resulted in the identification of >20,000 peptides from human plasma derived from approximately 8,000 source proteins (48). Comparable numbers (32,970 peptides, derived from 11,305 source proteins) have also been identified in pleural effusion (54).

The classical MS acquisition method used for such studies is data-dependent acquisition (DDA) mass spectrometry, where abundant peptide sequences are obtained by searching the acquired mass spectra against existing protein databases (65, 66). However, such approaches usually suffer from a lack of reproducibility, and lower abundant peptide species are typically neglected. As a consequence, (DDA) mass spectrometry is increasingly replaced by the arguably superior data-independent acquisition (DIA) mass spectrometry, in which all peptides irrespective of their abundance are fragmented in the mass spectrometer (67–70). More recently, attempts to incorporate quantitative aspects into the historically purely qualitative field have gained traction, utilizing both label-free (71, 72) and label-based (73, 74) quantification approaches.

## Challenges and future perspectives

In previous studies, similar sHLA peptidomes were observed for individuals sharing the HLA allotypes. Indeed, it is important to recognize that the HLA region is the most polymorphic region in the human genome (22, 75) and that HLA allo- and haplotypes determine the sHLA immunopeptidome (33). As a consequence, an analysis of two different populations has a high possibility of yielding two different sHLA immunopeptidomes. Because of the polymorphism of the HLA gene, the discovery of universal HLA-bound peptides biomarker is challenging. However, precision biomarkers (based on HLA types) would be an option. Another possibility is studying peptides present by non-classical HLAs, such as HLA-E and G, which are less polymorphic.

In order for biomarkers to be implemented in the clinical settings, they have to undergo a thorough validation study against a large cohort of samples from both cancer patients and healthy donors. This poses a significant technical challenge on the throughput capabilities of existing immunopeptidomics workflows, as emphasized by the Human Immunopeptidome Project (HIPP) (69). Many approaches have been attempted to overcome this challenge ranging from the use of multiplexing assays (73, 74) to establishing a 96-well format workflow applicable to both cell lines and tumor tissues (64). A promising recent study by Zhang and colleagues successfully incorporated the use of an automated liquid handling instrument (Assay MAP Bravo platform; Agilent Technologies) (76), which resulted in an overall improvement in speed, sensitivity, and also reproducibility. This automation has indeed created a standardized high-throughput workflow that eliminates human error and might pave the trend for future studies.

Another important challenge that has been plaguing immunopeptidomic studies is the amount of starting material needed. Considering the low abundance of sHLA-peptide complexes in body fluids, approximately 3-5 mL of plasma (33, 56) and at least 10 mL of pleural effusion (54) are required for each analysis. In fact, some TAA-derived peptides are only found in a higher volume of starting material (54), posing a risk of losing valuable biomarkers when working with a lower volume. While the implementation of IP enrichments has allowed for significant improvement in peptide recovery, it comes with its limitation for reliable clinical biomarker screening. Varying quantities of peptides (from as low as 17.5% (77) up to 99% (78)) have been observed to be lost in the process, possibly due to the varying IP conditions employed in the literature (64). Therefore, the development of a standardized protocol for this method is paramount.

Lastly, all the current studies on sHLA immunopeptidomes used conventional proteome databases and only considered intact (not biologically modified) sequences which are present in the human reference proteome (79). However, recent studies suggest the high prevalence of post-translationally modified peptides (such as phosphorylated, deamidated and glycosylated peptides) as well as proteasomally spliced epitopes in the immunopeptidome (80–83). On the other hand, the rise of proteogenomic studies, which combine genomic and proteomic approaches, will accelerate the expansion of current databases with novel and variant peptide species (84–86). Taken together, these breakthroughs have created and will create superior approaches to investigate HLA immunopeptidomes at an unprecedented level, further elevating its potential as a repertoire for cancer biomarkers.

## Conclusion

In summary, sHLA immunopeptidomes are a viable source of cancer biomarkers. sHLA complexes are present in most body fluids (31, 32, 34, 35, 37–42) and their peptide cargo contains many validated CTAs and TAAs (33, 46–48, 54). These peptides have the potential to be used as diagnostic, prognostic and/or predictive biomarkers for different cancer immunotherapy strategies such as immune checkpoint inhibitors, cancer vaccines and T-cell therapies. Continuous improvements in mass spectrometric instrumentation, bioinformatics and sample preparation over the past decade have allowed for more robust and comprehensive sHLA immunopeptidomics (57, 76, 77, 87, 88). However, we have to be acutely aware that there are currently very limited studies on liquid biopsy samples and a myriad of technical challenges to be overcome before its routine implementation in clinical settings (69). At this stage, developing standardized protocols for sHLA-based biomarker research would be an important initial step to ensure analytical validity and quality of future studies.

## Author contributions

ET designed and drafted the manuscript. RS and PF critically revised the manuscript and contributed to writing the final manuscript. All authors contributed to the article and approved the submitted version.
